# Supplementary material for: Bone marrow imaging reveals the migration dynamics of neonatal hematopoietic stem cells
Source: Commun Biol. 2022 Aug 2;5:776. doi: 10.1038/s42003-022-03733-x (PMC9346000; doi:10.1038/s42003-022-03733-x)
Supplement: Supplementary file 2 — Supplementary Information [file 42003_2022_3733_MOESM2_ESM.pdf]

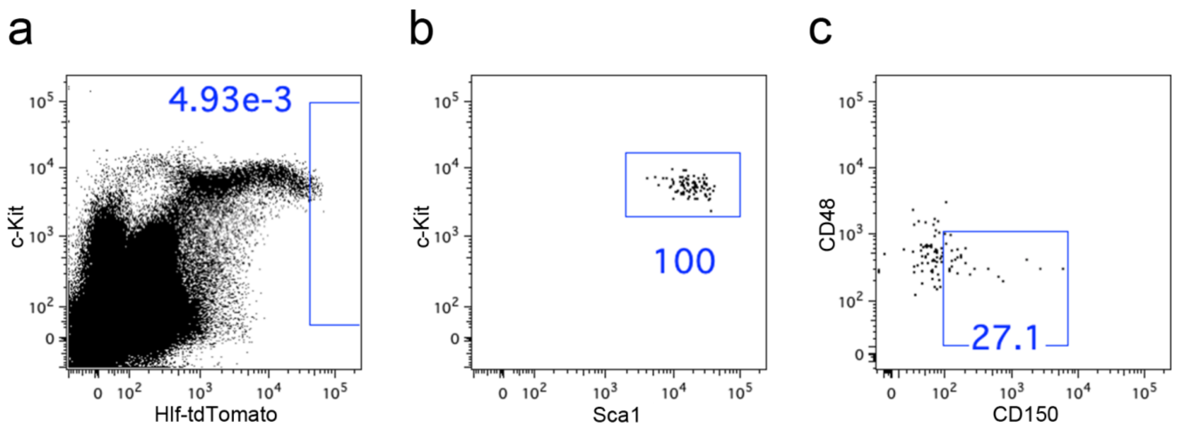

Supplementary Fig. 1.

(a) Flow cytometry analysis of Hlf-tdTomato^{hi} cells.

(b) All Hlf-tdTomato^{hi} cells expressed c-Kit and Sca1.

(c) CD48 and CD150 expression in Hlf-tdTomato^{hi} cells.

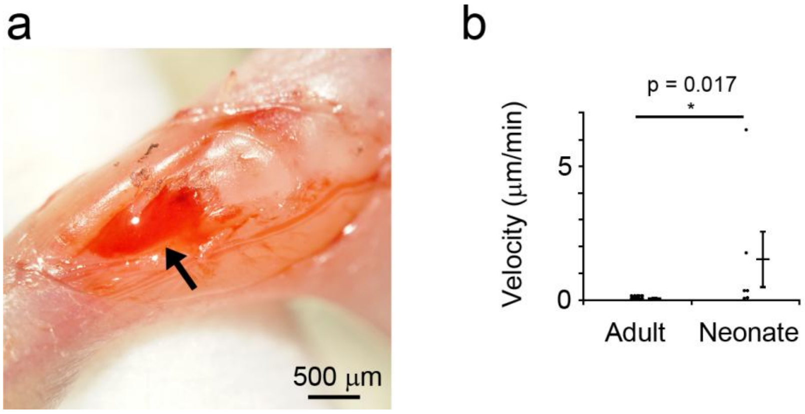

Supplementary Fig. 2.

(a) Bleeding from the neonatal tibia due to drilling (arrow). See also Supplementary Movie 1.

(b) Quantitative comparison of Hlf-tdTomato^{hi} cell dynamics between adults and neonates (Adult: ten cells from five mice; Neonate six cells from three mice).

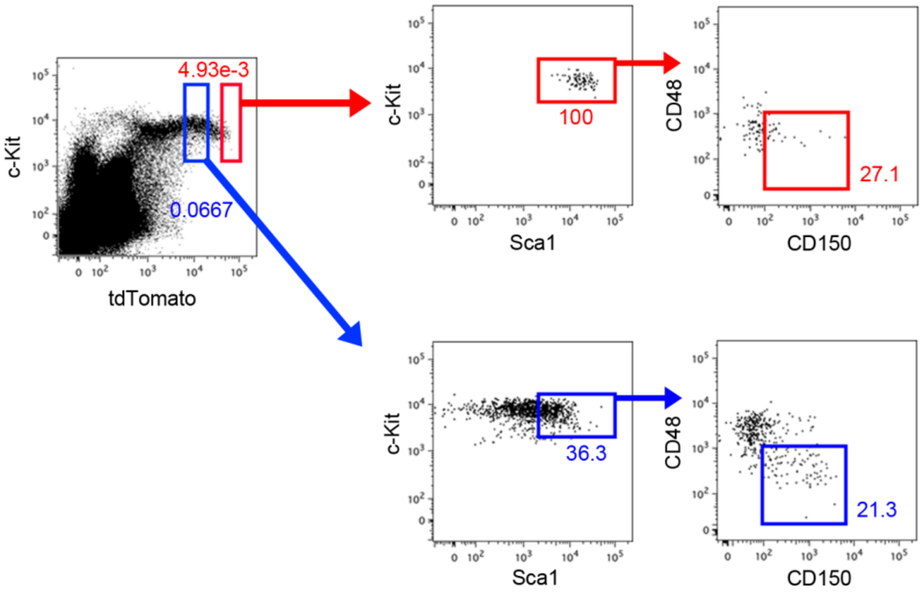

Supplementary Fig. 3.

Comparison of stem cell marker expression between Hlf-tdTomato^{hi} cells (red) and Hlf-tdTomato^{low} cells (blue).

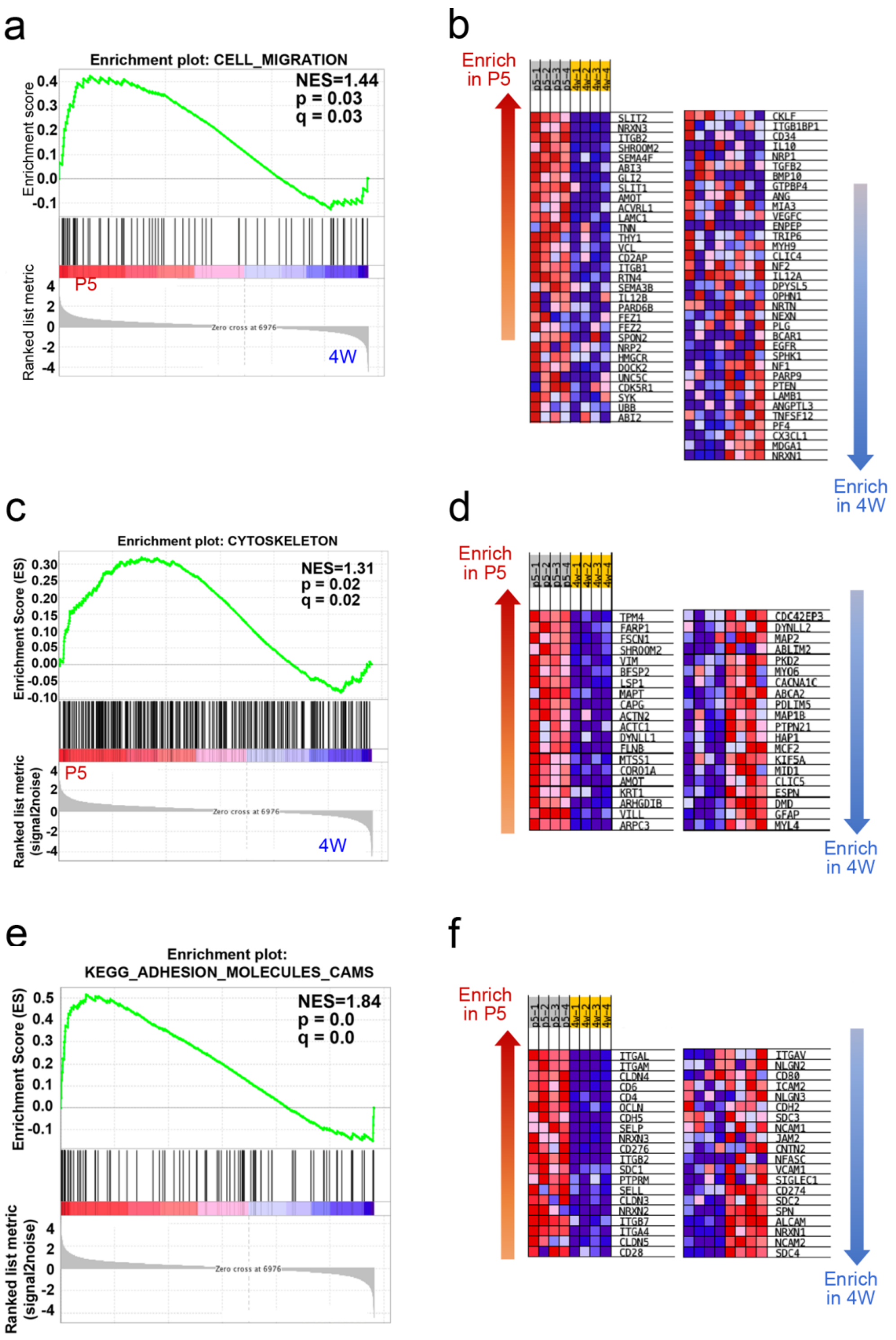

Supplementary Fig. 4.

Enrichment of gene set CELL_MIGRATION (a), CYTOSKELETON (c), and ADHESION MOLECULES (e) within differentially regulated genes in P5 neonatal and 4-week-old (4W) mouse HSCs. NES: normalized enrichment score. Genes enriched in P5 and 4W mouse HSCs. (b) CELL_MIGRATION, (d) CYTOSKELETON, and (f) ADHESION MOLECULES.

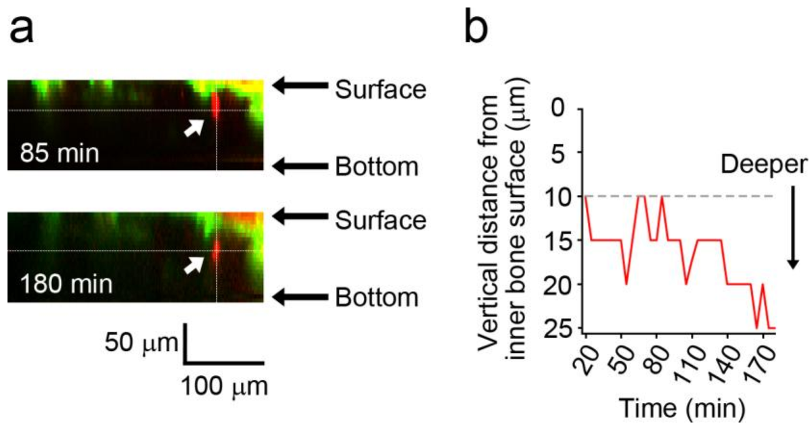

Supplementary Fig. 5.

(a) Orthogonal view of an Hlf-tdTomato^{hi} cell captured by intravital imaging of the tibial bone cavity of neonates where the blood vessels penetrating the bone are located. White arrows indicate Hlf-tdTomato^{hi} cell.

(b) Example measurements of the vertical distance between Hlf-tdTomato^{hi} cells and the inner bone surface in the neonatal tibia.

Supplementary Movie 1.

Bleeding from the neonatal (P2) tibia due to drilling.

Supplementary Movie 2.

Sequential Z-stack images (optical section thickness, 5 μm; 31 optical sections) were obtained via *in vivo* imaging of the bone marrow in the undrilled tibia of adult Hlf-tdTomato KI mouse.

Supplementary Movie 3.

Time-lapse Z-stack movie (2 h, stacks were obtained every 4 min) obtained via *in vivo* imaging of the bone marrow in the undrilled tibia of the adult Hlf-tdTomato KI mouse. Qtracker 655 signals (blue) gradually decreased due to hours of intravital imaging.

Supplementary Movie 4.

Sequential Z-stack images (optical section thickness, 5 μm; 32 optical sections) were obtained via *in vivo* imaging of the bone marrow in the undrilled tibia of the P2 Hlf-tdTomato KI mouse.

Supplementary Movie 5.

Time-lapse Z-stack movie (1.5 h, stacks were obtained every 4.5 min) obtained via *in vivo* imaging of the bone marrow in the undrilled tibia of the P2 Hlf-tdTomato KI mouse. Qtracker 655 signals (blue) gradually decreased due to hours of intravital imaging.

Supplementary Movie 6.

Time-lapse Z-stack movie (2.5 h, stacks were obtained every 5 min) obtained via *in vivo* imaging of the undrilled tibia of the P2 Hlf-tdTomato KI mouse showing rapid migration of tdTomato^{hi} cell in the bone cavity.
