# Supplementary material for: Bone marrow imaging reveals the migration dynamics of neonatal hematopoietic stem cells
Source: Commun Biol. 2022 Aug 2;5:776. doi: 10.1038/s42003-022-03733-x (PMC9346000; doi:10.1038/s42003-022-03733-x)
Supplement: Supplementary file 3 — Description of Additional Supplementary Files [file 42003_2022_3733_MOESM3_ESM.pdf]

Description of Additional Supplementary Files

File name: Supplementary Movie 1
Description: Bleeding from the neonatal (P2) tibia due to drilling

File name: Supplementary Movie 2
Description: Sequential Z-stack images (optical section thickness, 5 µm; 31 optical sections) were obtained via in vivo imaging of the bone marrow in the undrilled tibia of adult Hlf-tdTomato KI mouse.

File name: Supplementary Movie 3
Description: Time-lapse Z-stack movie (2 h, stacks were obtained every 4 min) obtained via in vivo imaging of the bone marrow in the undrilled tibia of the adult Hlf-tdTomato KI mouse. Qtracker 655 signals (blue) gradually decreased due to hours of intravital imaging.

File name: Supplementary Movie 4
Description: Sequential Z-stack images (optical section thickness, 5 µm; 32 optical sections) were obtained via in vivo imaging of the bone marrow in the undrilled tibia of the P2 Hlf-tdTomato KI mouse.

File name: Supplementary Movie 5
Description: Time-lapse Z-stack movie (1.5 h, stacks were obtained every 4.5 min) obtained via in vivo imaging of the bone marrow in the undrilled tibia of the P2 Hlf-tdTomato KI mouse. Qtracker 655 signals (blue) gradually decreased due to hours of intravital imaging.

File name: Supplementary Movie 6
Description: Time-lapse Z-stack movie (2.5 h, stacks were obtained every 5 min) obtained via in vivo imaging of the undrilled tibia of the P2 Hlf-tdTomato KI mouse showing rapid migration of tdTomatohi cell in the bone cavity.
