# Supplementary material for: Bone marrow imaging reveals the migration dynamics of neonatal hematopoietic stem cells
Source: Commun Biol. 2022 Aug 2;5:776. doi: 10.1038/s42003-022-03733-x (PMC9346000; doi:10.1038/s42003-022-03733-x)
Supplement: Supplementary file 10 — Reporting Summary [file 42003_2022_3733_MOESM10_ESM.pdf]

Corresponding author(s): Hidenobu Mizuno

Last updated by author(s): Jun 21, 2022

Reporting Summary

Nature Research wishes to improve the reproducibility of the work that we publish. This form provides structure for consistency and transparency in reporting. For further information on Nature Research policies, see our Editorial Policies and the Editorial Policy Checklist.

Statistics

For all statistical analyses, confirm that the following items are present in the figure legend, table legend, main text, or Methods section.

n/a	Confirmed	
☐	☒	The exact sample size (*n*) for each experimental group/condition, given as a discrete number and unit of measurement
☐	☒	A statement on whether measurements were taken from distinct samples or whether the same sample was measured repeatedly
☐	☒	The statistical test(s) used AND whether they are one- or two-sided<br>*Only common tests should be described solely by name; describe more complex techniques in the Methods section.*
☒	☐	A description of all covariates tested
☐	☒	A description of any assumptions or corrections, such as tests of normality and adjustment for multiple comparisons
☐	☒	A full description of the statistical parameters including central tendency (e.g. means) or other basic estimates (e.g. regression coefficient) AND variation (e.g. standard deviation) or associated estimates of uncertainty (e.g. confidence intervals)
☐	☒	For null hypothesis testing, the test statistic (e.g. F, t, r) with confidence intervals, effect sizes, degrees of freedom and P value noted<br>*Give P values as exact values whenever suitable.*
☒	☐	For Bayesian analysis, information on the choice of priors and Markov chain Monte Carlo settings
☒	☐	For hierarchical and complex designs, identification of the appropriate level for tests and full reporting of outcomes
☐	☒	Estimates of effect sizes (e.g. Cohen's d, Pearson's r), indicating how they were calculated

Our web collection on statistics for biologists contains articles on many of the points above.

Software and code

Policy information about availability of computer code

Data collection	FACS data were collected using FlowJo software (BD). Microscopic images were collected using LasX software (Leica).
Data analysis	For FACS analysis, FlowJo software (BD) was used. For RNA expression analysis, GSEA software (v3.0, Broad Institute) was used. For statistical analysis, Microsoft excel, R, and SPSS software (IBM) were used.

For manuscripts utilizing custom algorithms or software that are central to the research but not yet described in published literature, software must be made available to editors and reviewers. We strongly encourage code deposition in a community repository (e.g. GitHub). See the Nature Research guidelines for submitting code & software for further information.

Data

Policy information about availability of data

All manuscripts must include a data availability statement. This statement should provide the following information, where applicable:

- Accession codes, unique identifiers, or web links for publicly available datasets
- A list of figures that have associated raw data
- A description of any restrictions on data availability

Data are available upon reasonable request to the corresponding author.

Field-specific reporting

Please select the one below that is the best fit for your research. If you are not sure, read the appropriate sections before making your selection.

☒ Life sciences ☐ Behavioural & social sciences ☐ Ecological, evolutionary & environmental sciences

For a reference copy of the document with all sections, see nature.com/documents/nr-reporting-summary-flat.pdf

Life sciences study design

All studies must disclose on these points even when the disclosure is negative.

Sample size	The required sample size (N) was calculated by R3.6.3 using power.t.test function with the following arguments: sig.level = 0.05, power = 0.8, d =1, and SD=1 (d = 1, SD= 1 were used to set the effect size as 1). For some of the intravital imaging data, sample number was lower than the required sample size due to the technical difficulty.
Data exclusions	No data were excluded.
Replication	The reproducibility of the findings was examined in all of the experiments, excluding some of the intravital imaging experiments due to the experimental difficulty.
Randomization	Allocation was random in all of the experiments.
Blinding	Blinding was impossible in all of the experiments.

Reporting for specific materials, systems and methods

We require information from authors about some types of materials, experimental systems and methods used in many studies. Here, indicate whether each material, system or method listed is relevant to your study. If you are not sure if a list item applies to your research, read the appropriate section before selecting a response.

Materials & experimental systems

n/a	Involved in the study	
☐	☒	Antibodies
☒	☐	Eukaryotic cell lines
☒	☐	Palaeontology and archaeology
☐	☒	Animals and other organisms
☒	☐	Human research participants
☒	☐	Clinical data
☒	☐	Dual use research of concern

Methods

n/a	Involved in the study	
☒	☐	ChIP-seq
☐	☒	Flow cytometry
☒	☐	MRI-based neuroimaging

Antibodies

Antibodies used	For flow cytometry, anti-CD45 antibody (30-F11, 1:100, BioLegend), anti-c-Kit antibody (2B8, 1:400, BioLegend), anti-CD45.1 antibody (A20, 1:200[illegible]BioLegend), and anti-CD45.2 antibody (104, 1:200, Biolegend) were used.<br>For immunohistochemistry, Rabbit anti-DsRed antibody (for tdTomato) (632496, 1:500, Takara Bio Clontech), Alexa Fluor 488-conjugated anti-rabbit IgG (H+L) antibody (711-545-152, 1:1000, Jackson ImmunoResearch), PE-conjugated CD45.1 antibody (110708, 1:100, BioLegend), PE-conjugated CD45.2 antibody (109808, 1:100, BioLegend), and Alexa Fluor 647-conjugated TER-119 antibody (116218, 1:100, BioLegend) were used.
Validation	Available in manufactures website using the above information.

Animals and other organisms

Policy information about studies involving animals; ARRIVE guidelines recommended for reporting animal research

Laboratory animals	Laboratory mice, C57BL6, both sex, pups (postnatal day 2-4) and adults (3 month) were used in the study.
Wild animals	N/A
Field-collected samples	N/A
Ethics oversight	All animal experiments were approved by the animal commitiee of Kumamoto University.

Note that full information on the approval of the study protocol must also be provided in the manuscript.

Flow Cytometry

Plots

Confirm that:

☒ The axis labels state the marker and fluorochrome used (e.g. CD4-FITC).

☒ The axis scales are clearly visible. Include numbers along axes only for bottom left plot of group (a 'group' is an analysis of identical markers).

☒ All plots are contour plots with outliers or pseudocolor plots.

☒ A numerical value for number of cells or percentage (with statistics) is provided.

Methodology

Sample preparation	Mouse blood cells from the long bones were used.
Instrument	FACSAria IIIu (BD) was used.
Software	FlowJo software (BD) was used.
Cell population abundance	Cell population abundance was checked by re-sorting as necessary.
Gating strategy	Cells were gated for size exclusion (FSC-A/SSC-A) followed by doublet exclusion (FSC-H/FSC-W and SSC-H/SSC-W). Boundaries between negative and positive were determined by unstained and single stained control.

☒ Tick this box to confirm that a figure exemplifying the gating strategy is provided in the Supplementary Information.
